# Hepatic transcript signatures predict atherosclerotic lesion burden prior to a 2-year high cholesterol, high fat diet challenge

**DOI:** 10.1371/journal.pone.0271514

**Published:** 2022-08-04

**Authors:** Sobha Puppala, Kimberly D. Spradling-Reeves, Jeannie Chan, Shifra Birnbaum, Deborah E. Newman, Anthony G. Comuzzie, Michael C. Mahaney, John L. VandeBerg, Michael Olivier, Laura A. Cox

**Affiliations:** 1 Center for Precision Medicine, Wake Forest School of Medicine, Winston-Salem, North Carolina, United States of America; 2 Texas Biomedical Research Institute, San Antonio, Texas, United States of America; 3 The Obesity Society, Silver Spring, Maryland, United States of America; 4 South Texas Diabetes and Obesity Institute and Department of Human Genetics, The University of Texas Rio Grande Valley School of Medicine, Brownsville, Texas, United States of America; University of Alberta, CANADA

## Abstract

The purpose of this study was to identify molecular mechanisms by which the liver influences total lesion burden in a nonhuman primate model (NHP) of cardiovascular disease with acute and chronic feeding of a high cholesterol, high fat (HCHF) diet. Baboons (47 females, 64 males) were fed a HCHF diet for 2 years (y); liver biopsies were collected at baseline, 7 weeks (w) and 2y, and lesions were quantified in aortic arch, descending aorta, and common iliac at 2y. Unbiased weighted gene co-expression network analysis (WGCNA) revealed several modules of hepatic genes correlated with lesions at different time points of dietary challenge. Pathway and network analyses were performed to study the roles of hepatic module genes. More significant pathways were observed in males than females. In males, we found modules enriched for genes in oxidative phosphorylation at baseline, opioid signaling at 7w, and EIF2 signaling and HNF1A and HNF4A networks at baseline and 2y. One module enriched for fatty acid β oxidation pathway genes was found in males and females at 2y. To our knowledge, this is the first study of a large NHP cohort to identify hepatic genes that correlate with lesion burden. Correlations of baseline and 7w module genes with lesions at 2y were observed in males but not in females. Pathway analyses of baseline and 7w module genes indicate EIF2 signaling, oxidative phosphorylation, and μ-opioid signaling are possible mechanisms that predict lesion formation induced by HCHF diet consumption in males. Our findings of coordinated hepatic transcriptional response in male baboons but not female baboons indicate underlying molecular mechanisms differ between female and male primate atherosclerosis.

## Introduction

Cardiovascular disease (CVD) is the leading cause of morbidity and mortality worldwide, and atherosclerosis is one of the most prevalent types of CVD [1,2]. Atherosclerotic plaques, a hallmark of atherosclerosis, develop through accumulation of lipids and fibrous elements in the intimal layer of medium size and large arteries [3]. CVD and atherosclerosis are influenced by a wide range of genetic and lifestyle factors, and a number of risk factors (e.g., inflammation, cholesterol intake, etc.) have been identified [4]. Additionally, the interaction between diet and genes underlying variation will be able to provide more in-depth mechanistic studies of the cellular and molecular underpinnings of these responses [4,5].

The liver is the main organ responsible for regulating lipid metabolism. Changes in liver function contribute to CVD through multiple metabolic pathways and by secretion of inflammatory chemokines and cytokines that enhance plaque formation and altered vascular tone [6]. Dietary cholesterol and hepatic cholesterol clearance are risk factors for atherosclerosis, which have been studied extensively in humans and experimental animals [4,7–11] to understand how these risk factors influence the development of atherosclerotic plaques in the vasculature. However, there is a significant gap in our knowledge regarding how hepatic molecular networks influence CVD risk and contribute to CVD development. No studies to date have investigated hepatic gene networks that influence atherosclerotic lesion development throughout a long-term dietary challenge, due to the challenges in collecting longitudinal liver samples from healthy individuals at multiple time points while also controlling dietary intake [12]. By using a nonhuman primate (NHP) model of CVD where diet is controlled and longitudinal liver biopsy collection is feasible, we can identify gene networks (modules) predictive of, or correlated with, atherosclerosis and its risk factors. Baboons (Papio hamadryas) have been used to study molecular mechanisms influencing risk of CVD due to extensive physiological, metabolic, and genetic similarities with humans [13]. Baboons, like humans, develop atherosclerosis in response to dietary cholesterol and fat, and show individual variation in the extent of total lesion burden [4,8–10]. Study of the liver response to a high cholesterol, high fat (HCHF) diet challenge, together with a detailed assessment of lesion formation over time, is a powerful approach to identify genes that are upstream players in atherogenesis rather than genes that are altered in response to pathological changes.

Recent studies have shown that individual genes do not work alone but often interact with each other and jointly affect human diseases [14,15]. In addition, variation in complex disease phenotypes is a function of variation in multiple genes responding to an environmental challenge such as diet. The goals of this study were to identify variation in the liver transcriptome predictive of, and correlated with, atherosclerotic lesions in baboons fed a HCHF diet for 2y, and to determine whether the response was sex-specific. Because the baboon life-span is approximately one-fourth of humans, this is equivalent to an 8-y controlled diet study in humans [4].

One hundred eleven baboons were fed a HCHF diet for 2y. Our study design included collecting liver biopsies prior to a HCHF challenge and at acute and chronic challenge timepoints and quantifying total lesion burden at 2y, allowing us to identify genetic variation predictive of lesions (baseline), and gene-by-diet variation correlated with lesions, i.e., acute gene by diet interactions (7w) and chronic gene-by-diet interactions (2y). To our knowledge, this is the first study in a large cohort of NHP to identify hepatic genes that correlate with lesions. Unbiased Weighted Gene Co-expression Network Analysis (WGCNA) of liver transcriptome data showed no overlap of gene modules correlated with lesions and LDLC, indicating different hepatic genes influence LDLC metabolism than total lesion burden. As we describe in detail below, no modules of genes were predictive of lesions in females, and only one module at 2y, which was enriched for genes in the fatty acid beta oxidation pathway, correlated with lesions. In males, three modules at baseline and one at 7w were predictive of lesions, and five modules at 2y correlated with lesions. We performed pathway and network analyses to nominate functional pathways and networks for module genes. In modules significantly associated with lesion burden, we identified not only module genes that have variants associated with atherosclerosis in the GWAS, but also novel transcripts whose expression levels are associated with atherosclerotic risk. Our findings of sex-specific hepatic networks correlated with lesion burden suggest sex-specific molecular networks influence primate atherosclerosis-risk.

## Materials and methods

### Ethics statement, study design and data collection

All animal procedures were reviewed and approved by the Texas Biomedical Research Institute’s (TBRI) Institutional Animal Care and Use Committee (IACUC). Southwest National Primate Research Center (SNPRC) facilities at TBRI and the animal use programs are accredited by Association for Assessment and Accreditation of Laboratory Animal Care International (AAALAC), operate according to all National Institutes of Health (NIH) and U.S. Department of Agriculture (USDA) guidelines, and directed by veterinarians (DVM). The SNPRC veterinarians made all animal care decisions. All animals were housed in group cages allowing them to live in their normal social groups with *ad libitum* access to food and water. Enrichment was provided on a daily basis by the SNPRC veterinary staff and behavioral staff in accordance with AAALAC, NIH, and USDA guidelines. For this study, we utilized a cohort of olive baboons (*Papio hamadryas*; Taxonomy ID 9557) maintained as part of the baboon colony at the SNPRC, located on the campus of TBRI, San Antonio, Texas. The baboons were raised and maintained on a basal diet, low in cholesterol and fat (LCLF), prior to the study initiation. Fresh serum samples were collected and stored in aliquots at -80 °C until analysis. After study completion, a SNPRC veterinarian euthanized animals using ketamine and pentobarbital followed by exsanguination in accordance with American Veterinary Medical Association (AVMA) guidelines [16]. Humane endpoint was confirmed by the TBRI IACUC protocol as lack of pulse, breathing, corneal reflex, and response to firm toe pinch, and inability of the veterinarian to hear respiratory sounds and heartbeat by stethoscope [4].

#### Animals and diet challenge

Details of the cohort are provided [4]. Briefly, 111 baboons (n = 47 females, 64 males) were maintained on a LCLF diet prior to beginning a 2y HCHF dietary challenge. All baboons in the study were fed daily and allowed to eat *ad libitum*. Samples and data were obtained at baseline, 7w, and at the end of 2y. Details of diet composition are cited [4]. Baboons ages ranged from 8.1y to 17.0y. Though the relative amount of cholesterol fed in baboons exceeded than human standard diet, our main objective is not to mimic the effects of diet on the cardiovascular health of humans but to use diet as a tool to induce atherosclerotic lesions so that studies can be conducted on development, severity and disease progression, especially in species with greater genetic, anatomic and physiological similarities than other animal models [4].

#### Liver biopsy collection

Liver biopsy samples were collected just prior to beginning the 2y HCHF diet challenge (while on LCLF diet), then at 7w of HCHF diet, and at the end of 2y HCHF diet challenge. Baboons were sedated with ketamine (10 mg/kg), given atropine (0.025 mg/kg) and intubated. Anesthesia was induced and maintained with isoflurane (1–2%). Biopsies were collected from the left lobe of the liver using Temno Evolution Needle, gauge 14 (CareFusion, San Diego, CA). During post biopsy recovery analgesia was provided in the form of Stadol, 0.15 mg/kg, bid, for 3 days and ampicillin, 25 mg/day for 10d.

#### Arterial lesions

Details of arterial lesion assessments are provided [4]. Baboons were humanely euthanized after 2 years on the HCHF diet using standard necropsy procedures as described [4]. Following dissection, preparation, lesions (fatty streaks and/or raised fibrous plaques) were visually identified and assessed in three major arteries (the aortic arch, thoracic section of the descending aorta, and the common iliac) as described in [4]. Total lesion burden was quantified by taking the sum of percent covered by fatty streaks or plaques from the three major arteries and was calculated as the sum of the extent of percentage of lesions/plaques in aortic arch, thoracic aorta and common iliac artery.

#### Fat ether extract (FEE) or crude fat

We quantified total fat content in liver biopsy samples by the ether extraction method. Approximately 1.0g of liver dried at 105°C in a forced air oven. The liver sample was crushed using a wooden applicator and placed in a fat extraction apparatus. Fat was extracted using petroleum ether and refluxing for 3 hours into tared beaker. The beaker was placed in 105°C oven, the sample dried for 30 minutes, and placed in desiccant and cooled for 45 minutes before determining the dried weight [17].

### Quantitative trait data

Circulating CVD risk factors, including lipids and lipoproteins, total serum cholesterol (TSC) and triglyceride (TG) concentrations, lipoprotein-related enzymes, and biomarkers of inflammation and oxidative stress, were previously published for this cohort [4].

### RNA isolation from liver

Approximately 20 mg of frozen liver was homogenized in 1ml RLT buffer (Qiagen) using a BeadBeater (BioSpec, Bartlesville, OK) with zirconia/silica beads. RNA was extracted from the homogenate and impurities removed using the RNeasy MinElute Kit (Qiagen Valencia, CA) according to manufacturer’s instructions. RNA quality was assessed spectrophotometrically using a NanoDrop^™^ 8000 (Thermo Fisher Scientific, Wilmington, DE). RNA was stored at −80°C.

### Whole-genome expression profiling

cRNA was synthesized and biotin labeled using Illumina TotalPrep RNA Amplification Kit (Ambion Inc., Austin, TX) according to manufacturer’s instructions. Briefly, total RNA was used for first- and second-strand cDNA synthesis followed by in vitro transcription to synthesize biotin-labeled cRNA. cRNA was quality checked and then hybridized to HumanHT-12 v4 Expression BeadChip (Illumina Inc., San Diego, CA). Baboon and human genomic sequence similarity ranges from 94–100% [18]. In previous work we have shown good hybridization to targets of ≥ 94% similarity [19]. In addition, we have shown in multiple publications that the majority of probes on the HumanHT BeadChip can be used effectively in baboons, with very few genes not providing quality signal [18–21]. Individual cRNA samples were used to interrogate each BeadChip. Gene expression was detected and cleaned using GenomeStudio software (Illumina Inc.) and filtered using quality score of 0.95. Data are available at NCBI, GEO accession number GSE139981. The raw transcript data for all samples (n = 333: 111 animals at 3 time points) were pre-processed identically for background correction and normalization. Only those transcripts whose detection levels equaled or exceeded 0.2 of the detection p-value 0.95 in at least 50% of the total samples were selected for further analyses. The filtered transcript data were quantile normalized and log_2_-transformed.

### Statistical analysis

WGCNA was developed specifically to analyze gene expression data and reduce multiple testing burden. WGCNA was performed according to the R package WGCNA protocol using recommended parameters (https://horvath.genetics.ucla.edu/html/CoexpressionNetwork/Rpackages/WGCNA/) [22]. In brief, bi-weight mid-correlation (bicor) which is insensitive to outliers was used to generate correlation matrix for all pair-wise genes. Next, threshold function was used to obtain the soft threshold (power = 12) to construct an adjacency matrix in accordance with a scale-free network. This adjacency matrix was then transformed into a topological overlap matrix to measure relative gene interconnectedness and proximity. Finally, gene co-expression modules were identified based on hierarchical clustering according to topology overlap. The major parameters were set with a deep‐split value of 2 to branch splitting and a minimum size cutoff of 30. Finally, highly correlated modules were merged based on a merging threshold set at a height cut-off of 0.25. Modules were considered significant if the correlation was ≥ 0.30 and p-value < 0.05. WGCNA has the advantage of allowing analysis of continuous traits without binning the data for arbitrary phenotypic cutoffs in an analysis because binning data into categories typically translates into losing power. However, the sample size of 47 females and 64 males in our study is considered large for animal models.

### Construction of module-trait relationships

The gene modules summarize the main patterns of variation. The first principal component represents the summary of the module and is referred to as the module eigengene (ME). The relationship between MEs and clinical traits including circulating CVD risk factors was assessed by Pearson correlation. The modules and clinical traits that showed significant correlation (P < 0.05 and ρ ≥ 0.30) were selected for further investigation. A heat map was used for visualization of the correlations of each module-trait relationship.

### Pathway and network enrichment analysis

After performing WGCNA to obtain gene modules, the expression values of genes in each significant module were ranked by total lesion burden in female and male baboons (Table 1).

**Table 1:** Total lesion burden* in the top and bottom 10 animals.

	Top 10		Bottom 10		
	Mean	SD	Mean	SD	p-val
Females	125.81	25.09	18.53	9.71	7.1x10^-10^
Males	92.878	23.05	6.54	2.81	2.3x10^-10^

*Sum percent area covered by fatty streaks or plaques.

The purpose of ranking by total lesion burden from the top 10 and bottom 10 animals was to assess directionality of pathways and networks enriched in the significant modules. Fold changes for all of the genes in each significant module were determined for the ranked expression values between the top 10 and bottom 10 animals. In our study All the expression values with fold changes from the significant modules were then imported to Ingenuity Pathway Analysis (IPA) software (Qiagen) for core analysis. Pathways were ranked by -log p-value. Right-tailed Fisher’s exact test was used to calculate a significant association between module genes and genes in canonical pathways [23]. A -log p-value ≥2.0 was considered to show significant association (p-value ≤ 0.05). We used an end‐of‐pathway (EOP) [24] approach in the selected modules to prioritize and identify key pathways that may regulate molecular mechanisms affecting baboon liver in response to HCHF diet. EOP is a step by step analysis of key pathway genes starting from the beginning to the EOP. According to EOP analysis, a biological pathway may be relevant if gene expression changes (i.e., activation or inhibition) at the beginning and end of the pathway are consistent with the changes in the phenotype [24]. By working upstream from the end of a pathway to each event in the pathway, it is possible to define the critical steps that are likely to be rate limiting in the pathway response to the challenge [24]. In the present study, we found gene expression profiles at the EOP were consistent with the overall pathway change for the significant modules at all timepoints.

IPA upstream functional analysis was used to predict the upstream transcriptional regulators from EOP expressed genes in a module. These predictions are based on the literature compiled in the Ingenuity Pathway Knowledge Base. An overlap p-value was computed based on significant overlap between genes in the data set and known targets regulated by the transcriptional regulator. The activation z-score algorithm was used to make predictions.

Causal network analysis (CNA) was used to connect upstream or master regulators to downstream differentially expressed genes, based on the expected directionality of expression from database predictions [25]. Causal networks were ranked by a combination of predicted activation/inhibition and p-value. A positive z-score of 2 denotes significant predictors of activation and a negative z-score of 2 as significant predictors of inhibition. A p-value of < 0.05 was considered to be statistically significant [25]. To know whether lesion module genes were associated with GWAS variants, we downloaded GWAS gene IDs from the human GWAS catalog for CVD related traits [26].

## Results

### Total lesion burden

In the present study, total lesion burden was quantified by taking the sum percent area covered by fatty streaks or plaques from the three major arteries. The values for the total lesion burden are expressed as percentages of area covered. Using a one-way ANOVA, we found significant sex differences between atherosclerotic lesions in females and males (females 65.06±39.18, males 42.10±30.39, p = 0.0007). The observation of greater atherosclerotic lesions in females than males is consistent with our previous study [4]. As a result of significant sex differences, we performed subsequent analyses for females and males separately.

### Fat ether extract (FEE)

We found that liver fat content did not correlate with total lesion burden (males: r = 0.08, p = 0.527 and females: r = -0.19, p = 0.210). The central question of our study presented here was whether modules of hepatic transcripts correlate with atherosclerotic lesion burden, and whether the altered gene expression may provide insights into the pathophysiology. Due to the absence of any correlation of liver fat content with lesion burden, we did not include these measures in the manuscript. A Pearson correlation was used to see if there is a significant correlation between lesions and FEE. We did not find significant correlations between atherosclerotic lesions and FEE in the males and females.

### Correlation of clinical traits with total lesions

Correlation analyses of clinical traits related to atherosclerosis risk versus lesions showed the greatest correlations for LDLC, LDLC-related traits, and total serum cholesterol (TSC) at all three timepoints. For LDLC and TSC, 7w correlations were greater in females than males, but 2y correlations were greater in males than females. HDLC-related measures at baseline were predictive of lesions when combining females and males. Lipoprotein-associated phospholipase A_2_ (Lp-PLA_2_) was sex-specific, only showing correlation in males. C-reactive protein (CRP), paraoxonase b (PONb) and paraoxonase (PONc), total antioxidant status (TASraw), and Von Willebrand factor protein (VWF P) were only weakly correlated with lesions (Table 2).

**Table 2:** Correlations of clinical traits with total lesion burden.

	Baseline			7w			2y		
Traits	Females	Males	Both	Females	Males	Both	Females	Males	Both
**HDLC related**									
dHDL	0.26	0.16	0.31**	0.08	0.16	0.16	0.1	0.08	0.09
HDLC	0.16	0.11	0.20*	0.06	-0.12	-0.01	-0.05	-0.02	-0.07
HDLCge	0.17	0.11	0.21*	-0.12	-0.12	-0.002	NA	NA	NA
HDLMED	0.21	0.2	0.28**	0.06	0.06	0.04	-0.003	0.04	0.06
APOA1	NA	NA	NA	0.03	0.03	0.02	-0.09	-0.03	-0.1
APOB	NA	NA	NA	0.47**	0.39**	0.50**	0.30*	0.5	0.43**
APOE	NA	NA	NA	0.30*	0.30*	0.35**	0.25*	0.25*	0.20*
**LDLC related**									
LDLC	0.42**	0.25*	0.45**	0.57**	0.52**	0.60**	0.41**	0.53**	0.49**
dLDL	0.36*	0.30*	0.40**	0.44**	0.29*	0.41**	NA	NA	NA
BETCge	0.44*	0.25*	0.46**	0.57**	0.52**	0.60**	0.43**	0.54**	0.50**
BETMED	0.32*	0.25*	0.36**	0.37*	0.29*	0.39**	NA	NA	NA
**Circulating lipids**									
TRIG	-0.12	-0.06	0.05	-0.18	-0.12	-0.02	0.03	-0.16	0.07
TSC	0.35*	0.29*	0.42**	0.57**	0.44**	0.56**	0.37*	0.44**	0.41**
**Lipoprotein related**									
Lp-PLA_2_	0.27	0.1	0.16	0.27	0.29*	0.30**	0.28	0.38**	0.25*
PONb	0.23	-0.06	0.25*	0.02	-0.18	0.09	0.21	-0.09	0.19*
PONc	0.21	-0.12	0.21*	0.09	-0.16	0.14	0.24	-0.03	0.23*
**Inflammation and Oxidative stress**									
TAS	0.04	-0.09	-0.01	0.37*	-0.17	0.1	0.24	-0.1	0.03
VWF P	NA	NA	NA	-0.29	0.09	-0.16	-0.25	0.11	-0.11
CRP	0.17	0.16	0.23*	-0.17	0.07	-2E-04	0.23	0.04	0.20*

*p-value < = 0.05.

**p-value < = 0.001.

#### Identification of modules of genes correlated with lesions and CVD-related clinical traits

**Fig 1:**
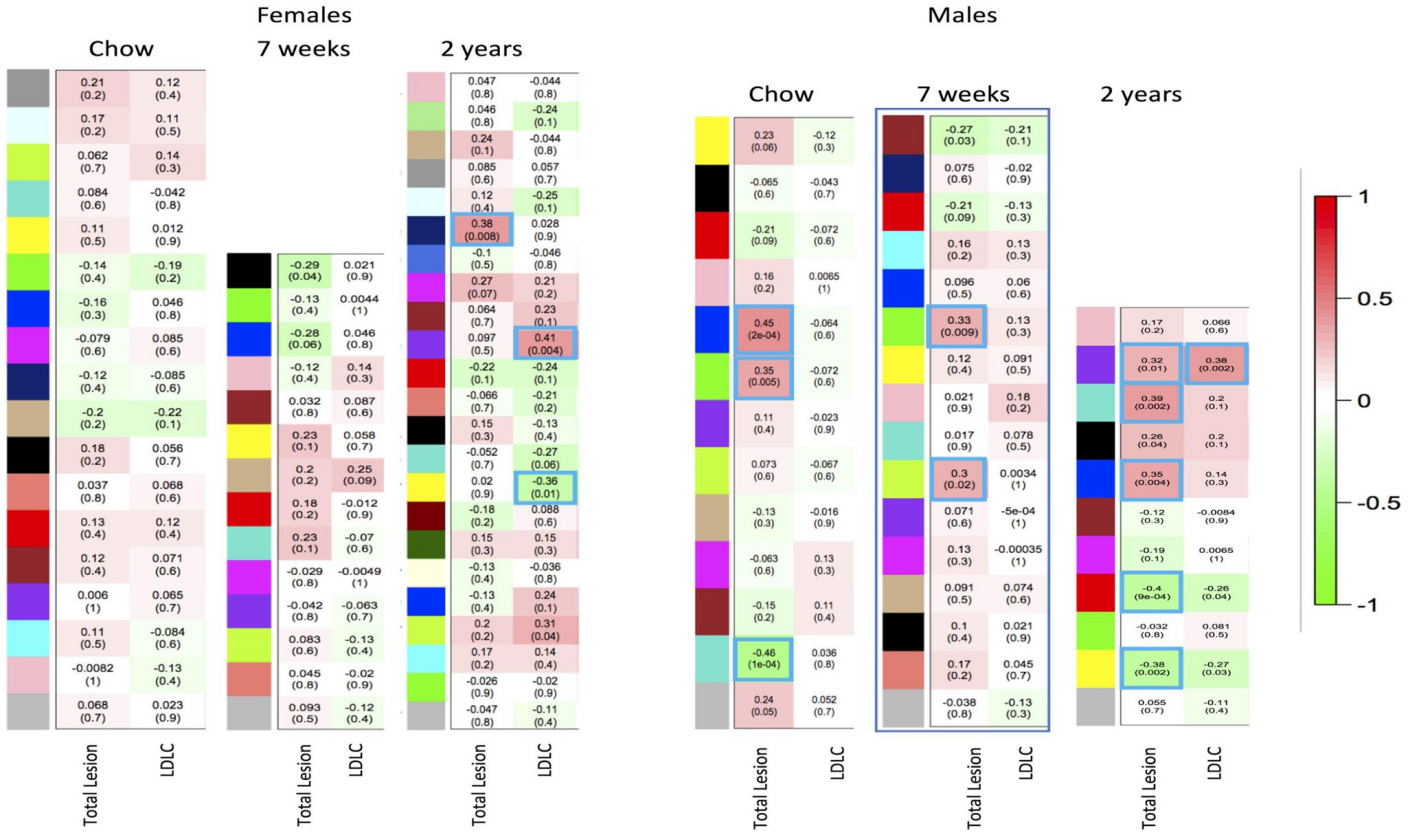
Modules for total lesion burden and LDLC. Each row corresponds to a module, the bottom column labels indicate total lesion burden or LDLC, and the top column label indicates timepoint. Numbers in each box indicate correlations with p values in parentheses. Positive correlations are indicated with red fill, negative with green fill, and significant modules are outlined blue.

A total of 15,139 transcripts at baseline, 15,325 transcripts at 7w HCHF, and 15,381 transcripts for 2y HCHF passed quality filters. WGCNA was used to identify modules of genes with correlated expression profiles, and to identify quantitative traits (e.g., lesions, LDLC, etc.) correlated with these modules. Modules correlated with total lesion and LDLC are shown in Fig 1. We found a strong clinical correlation between total lesion and LDLC at 2y but we did not find an overlap of gene networks between total lesion and LDLC at 2y. WGCNA is unbiased (gene function, knowledge base agnostic) and our findings of lack of overlap of genetic networks between total lesion and LDLC suggests that different hepatic genes influence LDLC metabolism and total lesion. Correlations for additional CVD-related traits are provided (S1–S6 Figs and S1 Table).

In females, one module was positively correlated with lesions at 2y (ρ = 0.38, p value = 0.008, n = 137 genes) and none at baseline or 7w (Fig 1 and S1–S3 Figs). Three modules were correlated with LDLC at 2y but did not overlap with the lesion module in females (S1 Fig). In males at baseline (S4 Fig), 3 modules correlated with lesions: blue (ρ = 0.45, p value = 0.0002, n = 1693 genes), green (ρ = 0.35, p value = 0.005, n = 700 genes) and turquoise (ρ = -0.46, p value = 0.0001, n = 1774 genes); at 7w (S5 Fig), 2 modules correlated with lesions: green (ρ = 0.33, p value = 0.009, n = 865 genes) and greenyellow (ρ = 0.30, p value = 0.02, n = 115 genes); and at 2y (S6 Fig), 5 modules correlated with lesions: turquoise (ρ = 0.39, p value = 0.002, n = 1691 genes), (purple ρ = 0.32, p value = 0.01, n = 78 genes), blue (ρ = 0.35, p value = 0.004, n = 1506 genes), red (ρ = 0.40, p value = 0.0009, n = 567 genes) and yellow (ρ = -0.38, p value = 0.002, n = 1390 genes). The purple (ρ = 0.38, p value = 0.002) module at 2y also correlated with LDLC (Fig 1 and S6 Fig).

Comparison of WGCNA and clinical correlations at 2y between females and males underscore the very discordant relationship of the liver transcriptome to lesion burden between sexes. In males, the purple module positively correlated with lesion burden and with multiple clinical measures related to CVD such as circulating LDL, TSC, and APOB (S6 Fig). These clinical measures also correlated with lesion burden (Table 2), indicating that hepatic genes associated with lesion burden are also directly related to clinical measures of CVD. Whereas, in females, the midnight blue module, which positively correlated with lesion burden, did not correlate with any clinical measures of CVD (S1 Fig) even though some of these measures correlated with lesion burden (Table 2). Furthermore, comparison of genes in modules for males that correlated with lesion burden (purple, turquoise, and blue) with genes in the female module (midnight blue) which positively correlated with lesion burden, shows an overlap of only 25 genes out of a total of 3275 genes (0.76%) in the 3 male modules and 137 genes (18%) in the female module.

### Pathway and network analysis of the 2y module genes correlated lesions in females

**Fig 2:**
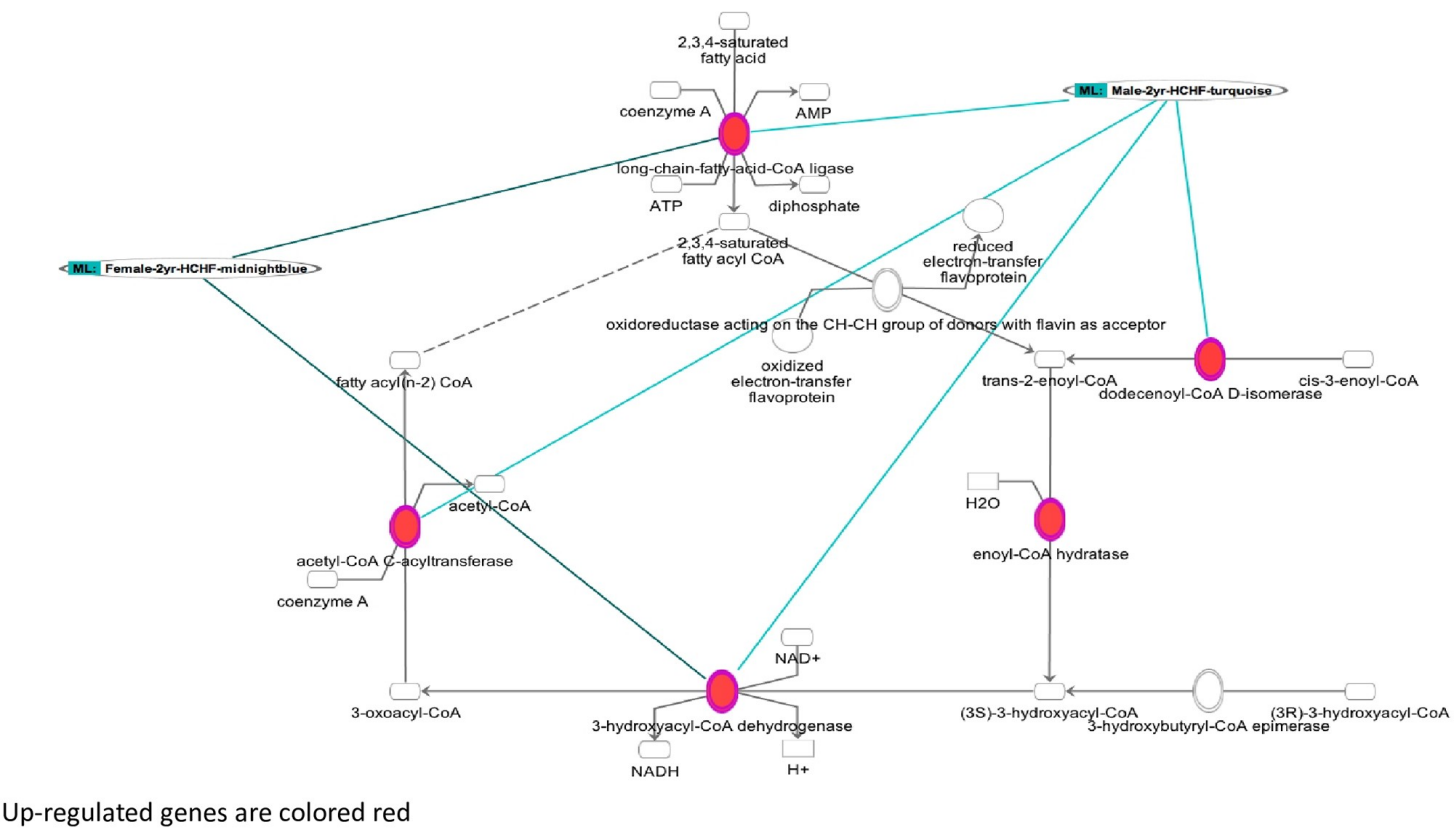
Fatty Acid β-Oxidation Pathway: Overlay of female midnight blue and male turquoise 2-year HCHF modules. Red fill indicates genes positively correlated with lesion burden. Green lines indicate genes from female and male modules respectively.

In females from the 2y timepoint, midnightblue module genes were enriched in 2 pathways: sulphate activation for sulfonation and fatty acid β oxidation (Table 3, Fig 2, S2A Table). The EOP gene for fatty acid β oxidation pathway included solute carrier family 27 member 5 (*SLC27A5/FATP5*) and hydroxyacyl-CoA dehydrogenase trifunctional multienzyme complex subunit beta (*HADHB*) genes which are upregulated with lesions at 2yr timepoint. Causal network analysis revealed *TP53* with 17 target genes positively correlated with lesions (Table 4 and S2A Table).

**Table 3:** Significant pathways passing end-of-pathway filter.

Ingenuity Canonical Pathways	-log(p-value)	Ratio
Female 2y HCHF midnightblue module		
Sulfate Activation for Sulfonation	2.02	0.50
Fatty Acid β Oxidation Pathway	1.98	0.06
Male baseline turquoise module		
EIF2 Signaling	7.51	0.16
Sirtuin Signaling Pathway	5.41	0.13
mTOR Signaling	4.35	0.13
Oxidative Phosphorylation	3.68	0.16
Protein Ubiquitination Pathway	3.49	0.11
Mitochondrial Dysfunction	3.33	0.13
Coagulation System	3.10	0.23
Serotonin Degradation	2.91	0.16
Adipogenesis Pathway	2.64	0.13
RAN Signaling	2.62	0.29
Iron Homeostasis Signaling Pathway	2.54	0.12
Male baseline blue module		
Ephrin Receptor Signaling	3.92	0.13
Phospholipase C Signaling	3.80	0.11
Cell Cycle Regulation by BTG Family Proteins	3.16	0.22
Estrogen Receptor Signaling	2.91	0.12
Agrin Interactions at Neuromuscular Junction	2.50	0.14
Nur77 Signaling in T Lymphocytes	2.37	0.15
T Cell Receptor Signaling	2.26	0.12
Oxidative Phosphorylation	2.21	0.12
Male 7w HCHF green module		
Opioid Signaling Pathway	3.10	0.07
CD28 Signaling in T Helper Cells	2.83	0.08
PI3K Signaling in B Lymphocytes	2.23	0.07
Male 2y HCHF turquoise module		
Thyroid Hormone Metabolism II (via Conjugation and/or Degradation)	3.95	0.23
Melatonin Degradation I	3.60	0.19
EIF2 Signaling	2.50	0.10
ERK/MAPK Signaling	2.14	0.10
Fatty Acid β Oxidation Pathway	2.08	0.19

**Table 4:** Regulatory networks with predicted activation state.

Upstream Regulator	Molecule Type	Predicted Activation State	Expr Fold Change	Activation z-score	p-value of overlap
Female 2y HCHF midnightblue module					
TP53	transcription regulator	Activated		1.8	7.61E-03
Male baseline turquoise module					
HNF4A	transcription regulator	Inhibited		-3.4	5.51E-16
MYC	transcription regulator	Inhibited		-4.2	4.64E-11
TP53	transcription regulator	Inhibited		-2.9	3.48E-09
HNF1A	transcription regulator	Inhibited		-4.1	1.25E-07
XBP1	transcription regulator	Inhibited	-1.05	-4.9	4.17E-05
RUVBL1	transcription regulator	Inhibited		-2.5	9.63E-05
KDM5A	transcription regulator	Activated		3.6	9.77E-05
TCF7L2	transcription regulator	Inhibited		-5.5	2.01E-04
NFE2L2	transcription regulator	Inhibited		-5.4	6.90E-04
PPARGC1A	transcription regulator	Inhibited		-2.5	8.65E-04
ESR1	ligand-dependent nuclear receptor	Inhibited		-3.2	1.30E-03
Male baseline blue module					
PTH	other	Activated		2.2	8.66E-05
SIM1	transcription regulator	Activated		4.3	2.85E-03
PPARGC1B	transcription regulator	Activated		3.0	4.60E-03
ARNT2	transcription regulator	Activated		4.1	4.82E-03
DDX17	enzyme	Activated		2.0	9.55E-03
TBXT	transcription regulator	Activated		2.0	1.79E-02
ESRRA	ligand-dependent nuclear receptor	Activated	1.022	2.4	1.91E-02
SMARCD3	transcription regulator	Activated		2.0	2.14E-02
Male 7w HCHF green module					
EGR2	transcription regulator	Activated		3.0	8.02E-03
Male 2y HCHF turquoise module					
HNF4A	transcription regulator	Activated		4.7	1.17E-11
TP53	transcription regulator	Activated		2.2	3.49E-08
NFE2L2	transcription regulator	Activated		4.3	2.81E-06
NR3C1	ligand-dependent nuclear receptor	Activated		2.2	4.57E-05
PPARA	ligand-dependent nuclear receptor	Activated		2.9	1.87E-04
MYC	transcription regulator	Activated		2.9	5.06E-04
SMAD3	transcription regulator	Activated		2.7	1.05E-03
FOXA2	transcription regulator	Activated		2.1	3.12E-03
TP63	transcription regulator	Activated		3.3	4.49E-03
FLI1	transcription regulator	Activated		2.2	4.77E-03
HNF1A	transcription regulator	Activated		3.3	5.37E-03
FOS	transcription regulator	Activated		2.6	6.20E-03
SMARCA4	transcription regulator	Activated		4.2	6.82E-03
TP73	transcription regulator	Activated		2.1	1.77E-02
CTNNB1	transcription regulator	Activated		2.9	1.93E-02
ETS2	transcription regulator	Activated		2.2	1.94E-02
SP1	transcription regulator	Activated		3.3	2.00E-02
ARNT	transcription regulator	Activated		2.4	2.10E-02
MTPN	transcription regulator	Activated	1.001	2.8	2.43E-02
HIF1A	transcription regulator	Activated		3.2	2.61E-02
XBP1	transcription regulator	Activated		2.7	2.62E-02
ATF2	transcription regulator	Activated	1.022	2.4	2.65E-02
ERG	transcription regulator	Activated		2.3	2.85E-02
ELF3	transcription regulator	Activated		2.2	2.98E-02
SMARCB1	transcription regulator	Activated		3.2	3.61E-02
NR1I3	ligand-dependent nuclear receptor	Activated		3.0	4.47E-02
Male 2y HCHF red module					
NR3C1	ligand-dependent nuclear receptor	Inhibited		-2.4	8.20E-04
PGR	ligand-dependent nuclear receptor	Inhibited		-2.9	3.03E-03
RELA	transcription regulator	Inhibited		-2.3	1.65E-02
CTNNB1	transcription regulator	Inhibited		-3.0	3.59E-02

### Pathway and network analysis of module genes correlated lesions in males

**Fig 3:**
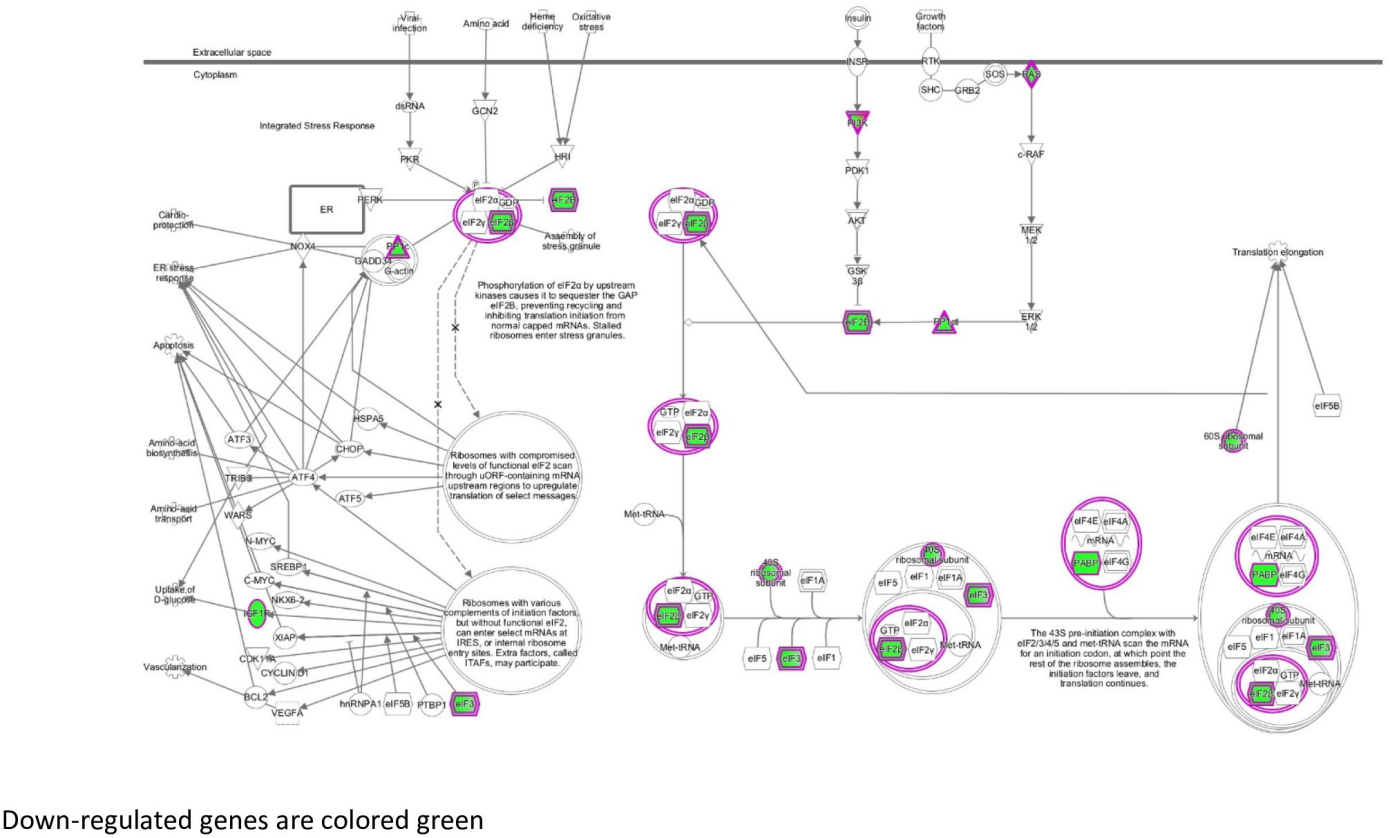
EIF2 signaling pathway: Male 2-year baseline module. Green fill indicates genes positively correlated with lesion burden.

In males at baseline, the turquoise module genes were enriched in 11 pathways and the blue module genes in 8 pathways. Among these pathways were EIF2 signaling (Fig 3), oxidative phosphorylation, ephrin receptor signaling, and estrogen signaling (Table 3, S2B and S2C Table). In addition, the turquoise and blue modules contained genes in 11 and 8 regulatory networks, respectively (Table 4, S2B and S2C Table). Master regulators in the networks from turquoise module genes included *HNF4A*, *MYC*, *TP53*, *HNF1A*, and *ESR1*, indicating overlap among pathways and causal networks at 2y (Table 4 and S2B Table). Master regulators in the networks from blue module genes included *PTH*, *SIM1*, *PPARGC1B*, *ARNT2*, and *ESRRA* (Table 3 and S2C Table).

**Fig 4:**
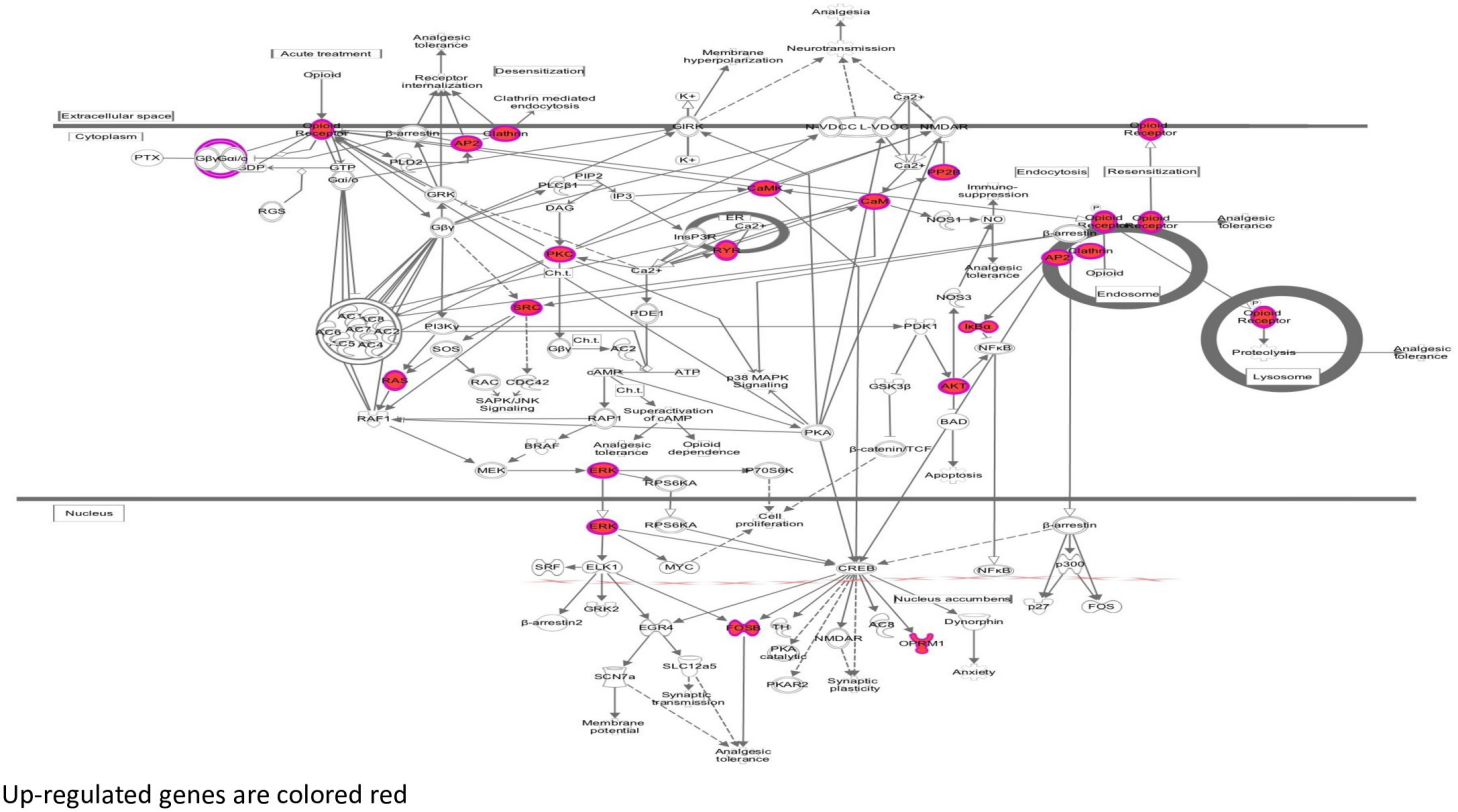
Opioid signaling pathway: Males 7-week green module. Red fill indicates genes positively correlated with lesion burden.

In males after 7w of HCHF diet, the green module included 3 pathways including opioid signaling (Fig 4, Table 3 and S2D Table), and one causal network with 10 genes targeted by the master regulator *EGR2* was observed (Table 4 and S2D Table).

**Fig 5:**
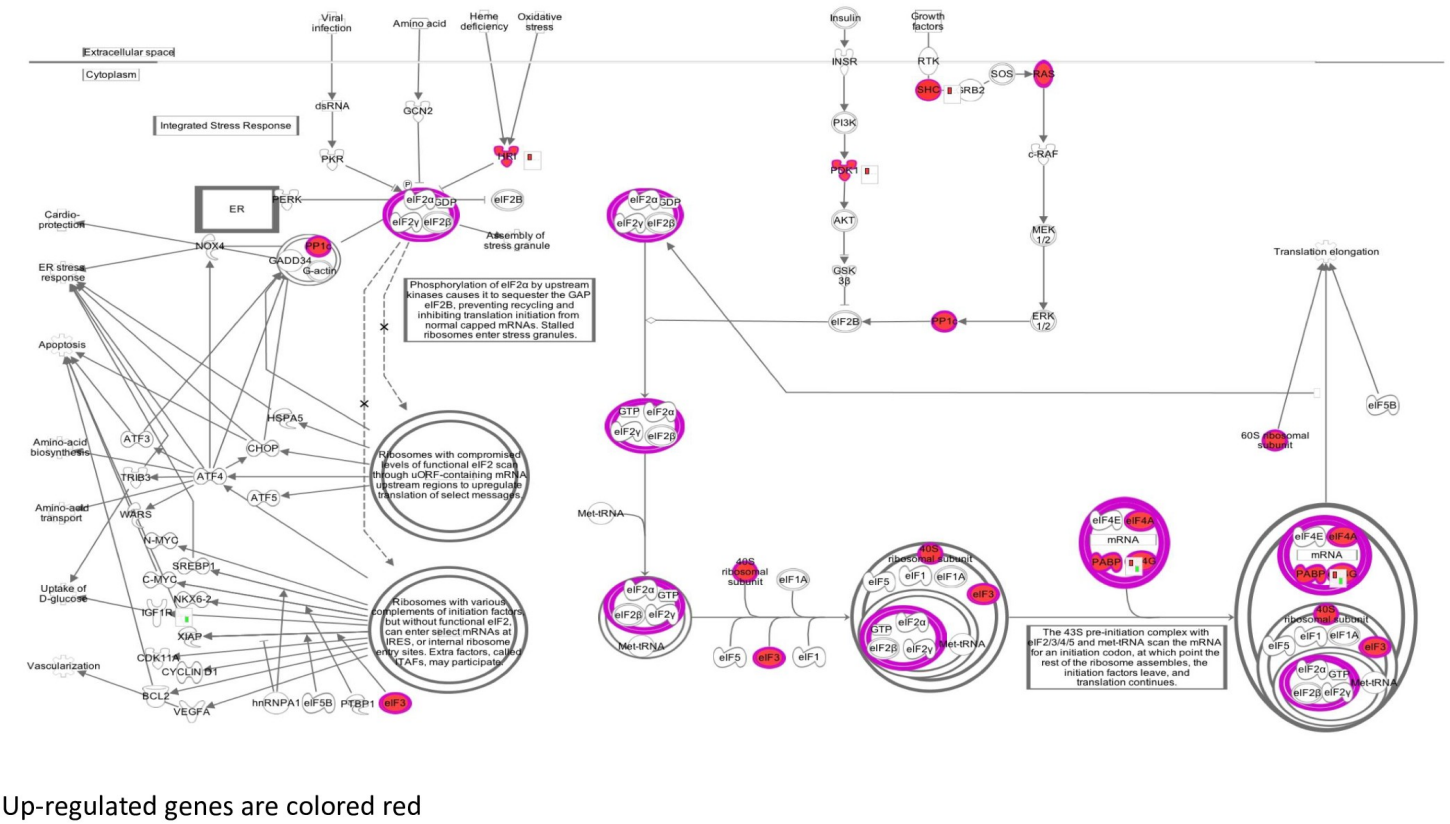
EIF2 signaling pathway: Male 2-year turquoise module. Red fill indicates genes positively correlated with lesion burden.

In males after 2y of HCHF diet, turquoise module contained 5 pathways including EIF2 Signaling (Fig 5, Table 3 and S2E Table) and fatty acid β oxidation (Fig 3, Table 3 and S2E Table); no pathways were enriched for genes from the red (Table 3 and S2F Table) and purple modules (Table 3 and S2G Table). Causal network analysis revealed 26 master regulators in the turquoise module (Table 4 and S2E Table) including *HNF4A*, *TP53*, *HNF1A* (Table 4 and S2E Table) and 4 in the red module including *NR3C1* (Table 4 and S2F Table). Causal network analysis revealed no regulators in the purple module.

### Comparison of genes in significant lesion modules human genes containing single nucleotide variants associated with CVD

Though GWAS variants do not fully relate with lesion module gene expression, it would be of great interest to know whether lesion module genes obtained in our study were previously reported to be associated with CVD related traits. To know whether lesion module genes were associated with GWAS variants, we downloaded GWAS gene IDs from the human GWAS catalog with CVD related trait annotations of coronary artery disease, carotid intima media thickness, C reactive protein, cholesterol total, HDL cholesterol, LDL cholesterol, and triglycerides. In our study, we found four genes with single nucleotide variants (SNVs) associated with coronary artery disease—*HNF1A*, *LOX*, *SMAD3*, *and SMARCA4*, and one gene, *EBF1*, with a SNV associated with carotid intima media thickness for the gens in significant modules. An additional 37 genes with SNVs were associated with LDL cholesterol, cholesterol total, and HDL cholesterol including *HNF1A*, *SMARCA4*, *APOE*, *PPARA*, *PPARG*, *HNF4A*, and *CDKN1A* (Table 5).

**Table 5:** Module genes with cardiovascular disease related human GWAS variants.

GWAS Trait	# Genes	Gene IDs
Coronary artery disease	4	HNF1A, LOX, SMAD3, SMARCA4
Carotid intima media thickness	1	EBF1
C reactive protein	10	APOE, ERCC1, ETS2, HNF1A, HNF4A, MAFB, ONECUT1, SPI1, TRPS1, ZGPAT
Cholesterol total	8	APOE, HNF1A, MAFB, NR1I2, PPARA, PPARG, SMARCA4, TRPS1
HDL cholesterol	8	APOE, GLIS3, NR1H3, PPARG, RUNX1, SPIC, THRB, TRPS1
LDL cholesterol	9	APOE, AR, HNF1A, MAFB, PPARG, SMARCA4, TAF4B, ZFHX3, ZFPM1
Triglycerides	2	APOE, CDKN1A

We selected the list of genes in significant modules that were not included in the GWAS CVD, significant pathways or networks. We found a total of 249 genes that fit these criteria with 30 or more genes in each module—50 of these genes were found in more than one module including CLOCK genes, homeobox genes, and zinc finger protein genes (Table 6 and S3 Table). We called these genes as potentially novel CVD associated genes.

**Table 6:** Module genes with no GWAS variants, and not found in any significant pathways or networks.

Sex	Timepoint	Module	# Genes
Female	2 year	Midnight blue	30
Male	Chow	Blue	40
Male	Chow	Turquoise	61
Male	7 week	Green	49
Male	7 week	GreenYellow	30
Male	2 year	Red	35
Male	2 year	Turquoise	85

249 unique genes; 50 Genes in >1 module.

## Discussion

The goal of this study was to identify hepatic genes that may contribute to lesion formation and progression of lesion burden in response to a HCHF diet, since the liver is a key organ in regulating lipid and cholesterol metabolism, and therefore may play a crucial role in affecting lesion formation under these dietary conditions. To our knowledge, this is the first study of a large NHP cohort to identify hepatic genes correlated with lesions. Our study design, which included collecting liver biopsies prior to a HCHF challenge and at acute and chronic challenge timepoints, allowed us to identify genetic variation predictive of lesions (baseline), and gene by diet variation correlated with lesions, i.e., acute gene by diet interactions (7w) and chronic gene by diet interactions (2y). The unbiased WGCNA approach was used to study the relationship between variation in hepatic transcripts with lesions, and clinical traits of lipid/lipoprotein metabolism and inflammation.

### Baseline diet: Hepatic gene expression and lesions

Overall, we found significant pathways in males at all three timepoints. At baseline, males showed enrichment of genes in EIF2 signaling, oxidative phosphorylation, ephrin receptor signaling and estrogen receptor signaling. Studies showed that downregulation of EIF2 signaling and oxidative phosphorylation promotes progression of atherosclerosis [27–30] and NASH which is an early marker of atherosclerosis [31–33]. Additionally, upregulation of ephrin receptor signaling implicate eph receptors and their ephrin ligands in this pathway as potential regulators of atherosclerosis [34]. Estrogen receptor signaling is also known to play an important role in CVD in both females and males [35–37]. Although estrogens are viewed as female sex hormones and androgens as male sex hormones, estrogens and androgens govern a number of physiological processes in both females and males [38]. Similarly, several endocrine factors, including androgens and estrogens, and biochemical factors are involved in the atherosclerotic process. The interaction between sex and CVD is affected by a plethora of extrinsic and intrinsic factors, and so may not be attributed to a single factor such one sex hormone alone.

According to the network analysis at baseline, the upstream regulators *HNF4A* and *HNF1A* are predicted to be inhibited in our study. *HNF4A* plays a critical role in in cholesterol, fatty acid and glucose metabolism [39]. Specifically, in the liver, *HNF4A* activates hepatic gluconeogenesis [40] and regulates the expression of several genes, including apolipoproteins [41]. Studies have shown that *HNF4A* is central to the pathogenesis of NASH and regulates the transcription of NAFLD progression genes [42]. *HNF4A* was also shown to regulate *HNF1A*, another transcription regulator involved in lipid and amino acid metabolism [42]. The inhibition of these upstream regulators at baseline correlated with increased lesions suggests that genetic variation in these genes is predictive of atherosclerotic lesions. The role of *HNF4A* and *HNF1A* in progression of atherosclerotic lesions needs further investigation.

### Acute diet challenge: 7w hepatic gene expression and lesions

In males, modules at 7w are enriched for genes in opioid signaling; liver expression of this pathway has not previously been shown to be associated lesions. Opioid receptors belong to family of inhibitory G-protein-coupled receptors [43] and are predominantly expressed in brain. The μ opioid receptor (*MOR*) is known to be expressed in peripheral tissues, including liver [44]. Although, the opioid system is known primarily for its pain-relieving and addictive properties, it has also been shown to play roles in control of energy consumption [45,46] and energy expenditure, and lipid [47] and glucose metabolism [48–51]. In our study, opioid signaling during the acute phase of the diet challenge is positively correlated with lesions, suggesting that *OPRM1* is involved in regulation of the dietary preference for fat [52], perhaps leading to lesion development. Correlation of opioid signaling with progression of atherosclerosis development in CVD is very interesting and requires further investigation to determine mechanistic role(s).

### Chronic diet challenge: 2y hepatic gene expression and lesions

At 2y, modules were enriched for genes in fatty acid β oxidation pathway in both females and males—the only overlap between females and males. The fatty acid β-oxidation pathway breaks down fatty acids to produce energy and regulates hepatic fatty acid uptake [53–55]. Khan-Merchant et al [55] suggested that oxidized fatty acids play an important role in oxidative stress, bile acid metabolism, lipid metabolism, and body weight regulation in mice. In females, the fatty acid β oxidation pathway included *SLC27A5* and *HADHB* genes; whereas, in males a more robust enrichment was found with genes *ACAA1*, *SLC27A2*, *HADHB*, *HSD17B4*, *ECI1*, *and HADH*, emphasizing the significant molecular differences between females and males.

The upstream master regulator in females at 2y is *TP53* which is an important regulator of cell proliferation and programmed cell death (apoptosis), and up-regulation of TP53 is associated with development of atherosclerosis [56] and inflammation in atherosclerotic coronary arteries [57]. This is a small network with only 10 genes compared with those found in the males at all 3 timepoints. However, 3 of these genes, *GLRX*, *SDHA and CSF3R*, are known to play key roles in hepatic liver metabolism [58] and inflammation [59] suggesting these genes play an important role in lesion development in female primates. Studies have shown that women have greater frequency of cardiovascular disease than men, including atherosclerosis with a heavier risk factor burden, even after adjusting for age [60]. Atypical symptoms of CVD are also more common in women than men with outcomes from chronic and acute coronary syndromes worse in women than in men. In addition, in-hospital mortality after acute myocardial infarction is higher among younger women compared to male peers [60]. Taken together, these findings highlight sex differences in early presentation of CVD and likely underlying mechanisms [60]. Our findings of greater lesion burden in female baboons are consistent with findings in humans.

Network analysis of males at 2y did not show any overlap of upstream regulators with females. The network regulator HNF4A was positively correlated with lesions at 2y indicating gene by diet interactions influencing variation in this network. The *HNF4A* network is composed of 35 target genes, including activation of *APOE* which contributes to altered VLDL metabolism and increased atherosclerosis susceptibility in NAFLD [61]. HNF4A also activated *APOC3*, which may contribute to hypertriglyceridemia [62]. *NFE2L2*, which was activated, was previously shown to activate *PPARG*, an important regulator in adipogenesis, lipid metabolism and atherosclerotic [63,64]. The network with regulator *NR3C1* was activated, inhibiting *TGFB3*, which is involved in the pathogenesis of atherosclerosis *via TGF-β* signaling [65]. In addition, *CTNNB1 was also activated* inhibiting *TGFB3* and is involved in the development of NAFLD [66]. Taken together, causal network analysis suggests multiple regulators contribute to lesion formation in males.

Comparison of genes within the female and male modules at 2y showed significant sex-differences. The one module in males that correlated with lesion burden also correlated with multiple CVD-related clinical measures. In contrast, the female module that correlated with lesion burden did not correlate with any CVD-related clinical measures. Comparison of genes in modules that correlated with lesion burden between females and males showed only a small number of genes overlapped—25 genes out of a total of 3275 genes (0.76%) in the 3 male modules. Although the overlap seems greater when looking at the female module (18%), this is due to the far fewer number of genes in the female module– 137 versus 3275 in males (24x greater). These findings indicate fundamental molecular differences in female versus male livers in relation to lesion burden, and suggest that the type of molecular response (transcriptional, post-transcriptional, translational, etc.) differs significantly. Based on these findings, it is unlikely that a single factor such as a hormone receptor or signaling molecule are driving these sex-specific differences, but rather these differences are due to very different complex molecular networks at multiple regulatory levels.

WGCNA also allowed us to identify genes correlated with total lesion burden that are not part of canonical pathways or annotated causal networks. Out of 249 genes in significant modules that were not enriched in pathways or networks, four genes, *LMNA a*nd *MMP12* at baseline, *NPY* and *RXRA i*n females and males at 2y have previously been shown to be associated with atherosclerosis [67,68]. The remaining 245 genes in these modules are potentially novel players in the genetics and epigenetics of atherosclerotic lesion initiation and development.

A secondary goal of this study was to evaluate genetic and epigenetic overlap in commonly used clinical measures of CVD risk with lesions. Previous analyses in this cohort showed association of lipoprotein measures with lesions [4]. In females, we found no overlap between CVD risk measures and lesion modules. In males, we only observed overlap at 2y with the purple module containing 78 genes. Two genes in this module, *ACLS3* [69–72] and *CETP* have previously been associated with CVD risk [73]. These findings suggest different hepatic genes influence LDLC metabolism than total lesion burden.

One hypothesis explaining the gene expression changes seen in our study could be that hepatic gene expression changes initially have a local effect on fat accumulation in the liver, and with this perturbation leads to increased lesion formation in the periphery. While we do not have imaging data allowing the assessment of ectopic fat levels in and around the liver or other organs, we assessed total fat content in liver biopsy samples as a surrogate measure of abnormal fat storage. No correlation to lesion burden was detected, suggesting that the transcriptional changes observed do not have an overt direct effect on fat storage in the liver itself, and rather seem to modulate peripheral lipid handling.

## Conclusions

We found female baboons had more variation in lesions than males. We did not find gene modules predictive of lesions or correlated with acute HCHF diet in females. However, in males, both predictive and acute HCHF diet modules correlating with lesions were found. Further underlying sex differences, only one module was identified in females after 2y HCHF diet; whereas, multiple modules and pathways and networks within these modules were identified in males. In addition, very few genes in these in these modules were common to females and males. Identification of pathways and genes previously associated with development and severity of atherosclerotic lesions, such as fatty acid β oxidation, validate the similarities between baboons and humans. In addition, this unique study design where we were able to capture acute molecular responses to the HCHF diet, which is not feasible in humans, revealed that opioid signaling at 7w is highly correlated with lesions in males, potentially linking consumption of HCHF diet and hepatic μ-opioid receptor activity. Our results suggest that transcriptional and post-transcriptional mechanisms contribute to atherosclerosis in males whereas in females, post-transcriptional appears to play the major role. All of the above, advance our understanding of pathways and genes predictive of atherosclerotic lesions prior to exposure and after acute exposure to a HCHF dietary challenge in primates. Of interest also are our findings of 245 genes in significant modules not previously associated with cardiovascular disease, which may provide novel information for atherosclerotic risk and require further investigation.

## Limitations

The goal of the study was to use an unbiased approach to identify molecular changes associated with diet and atherosclerotic risk. This approach allowed us to nominate networks and regulators of the networks that are central to hepatic response to a chronic HCHF dietary challenge. One limitation of the study is inclusion of proteomic data, with protein abundance and post translational modifications, to validate the regulatory networks. In future work, we will develop the necessary baboon specific reagents for network validation.

## Supporting information

S1 Fig Female 2-year lesion module trait relationships. (PDF)

S2 Fig Female 7-week lesion module trait relationships. (PDF)

S3 Fig Female baseline lesion module trait relationships. (PDF)

S4 Fig Male baseline lesion module trait relationships. (PDF)

S5 Fig Male 7-week lesion module trait relationships. (PDF)

S6 Fig Male 2-year lesion module trait relationships. (PDF)

S1 Table Modules and significant correlations with CVD-related traits. (XLSX)

S2 Table A. Females 2-year midnightblue module pathway and network passing EOP. B. Males baseline turquoise pathways and networks passing EOP. C. Males baseline blue pathways and networks passing EOP. D. Males 7-week green pathways and networks passing EOP. E. Males 2-year turquoise pathways and networks passing EOP. F. Males 2-year red pathways and networks passing EOP. G. Males 2-year purple pathways and networks passing EOP.
(XLSX)

S3 Table Module genes with no GWAS variants. (XLSX)
