# Supplementary figures and images for: Hepatic transcript signatures predict atherosclerotic lesion burden prior to a 2-year high cholesterol, high fat diet challenge
Source: PLoS One. 2022 Aug 4;17(8):e0271514. doi: 10.1371/journal.pone.0271514 (PMC9352111; doi:10.1371/journal.pone.0271514)

S1 Fig. Female 2-year lesion module trait relationships

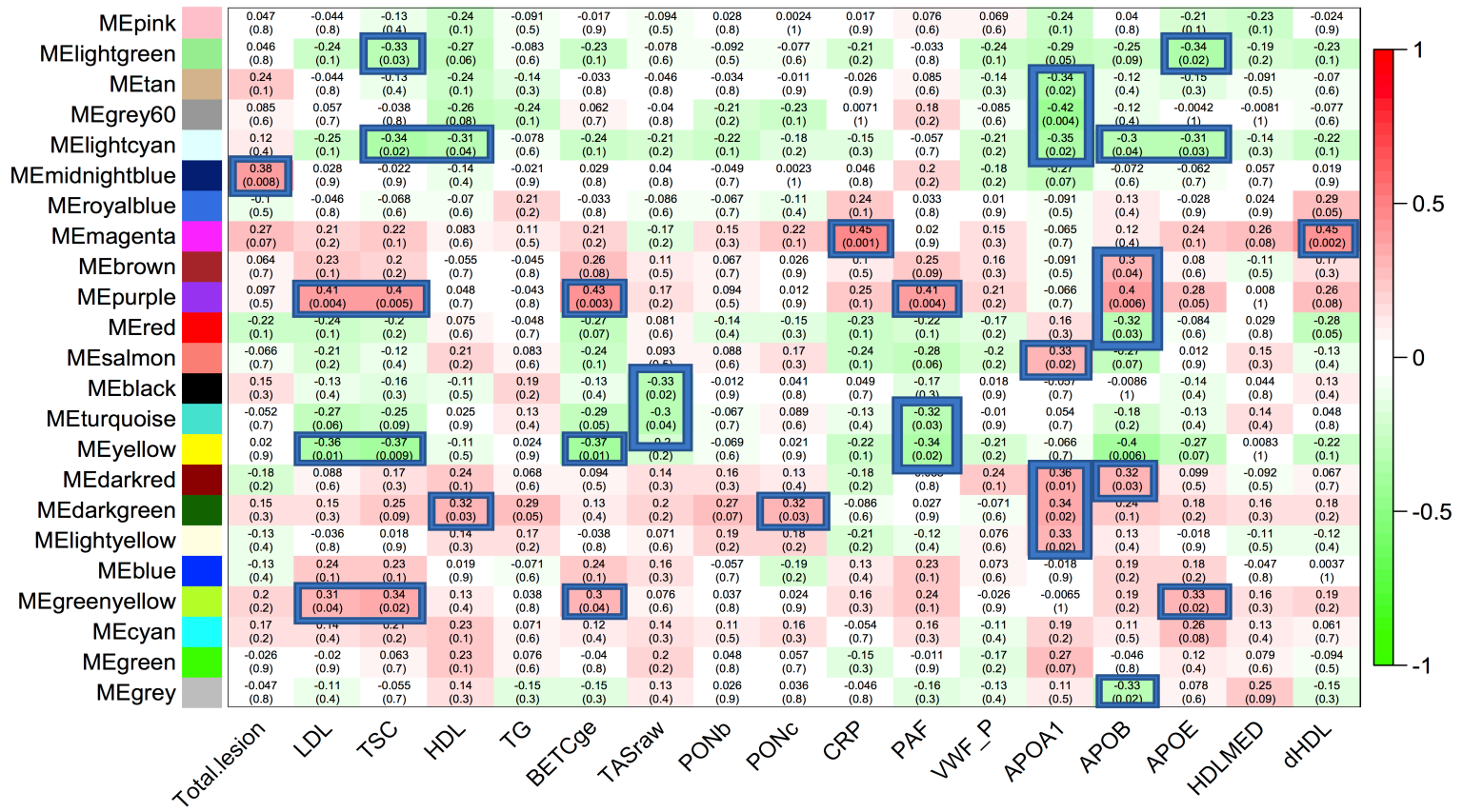

Supplement: S1 Fig — (PDF) [file pone.0271514.s001.pdf]

S2 Fig. Female 7-week lesion module trait relationships

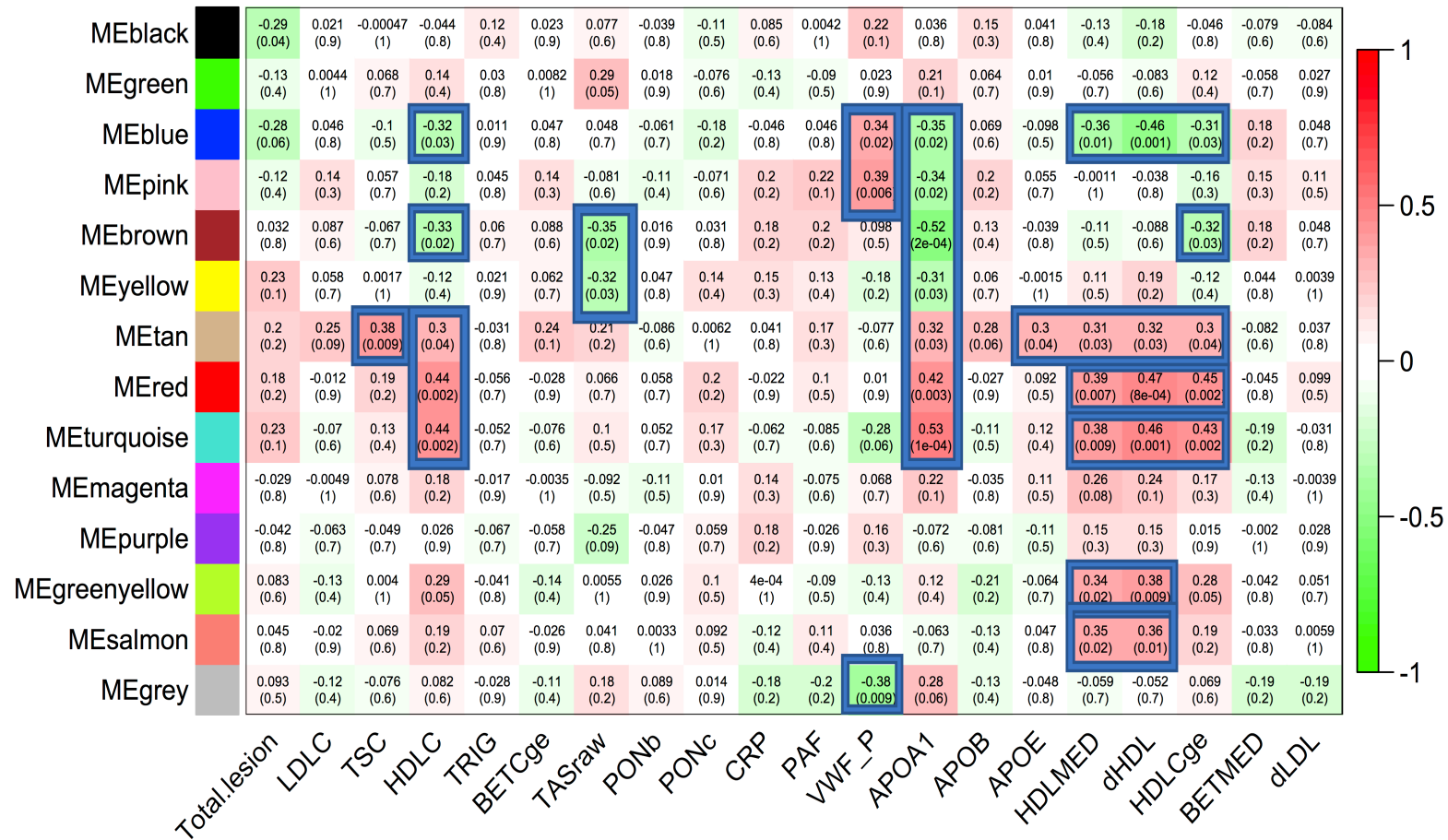

Supplement: S2 Fig — (PDF) [file pone.0271514.s002.pdf]

S3 Fig. Female baseline lesion module trait relationships

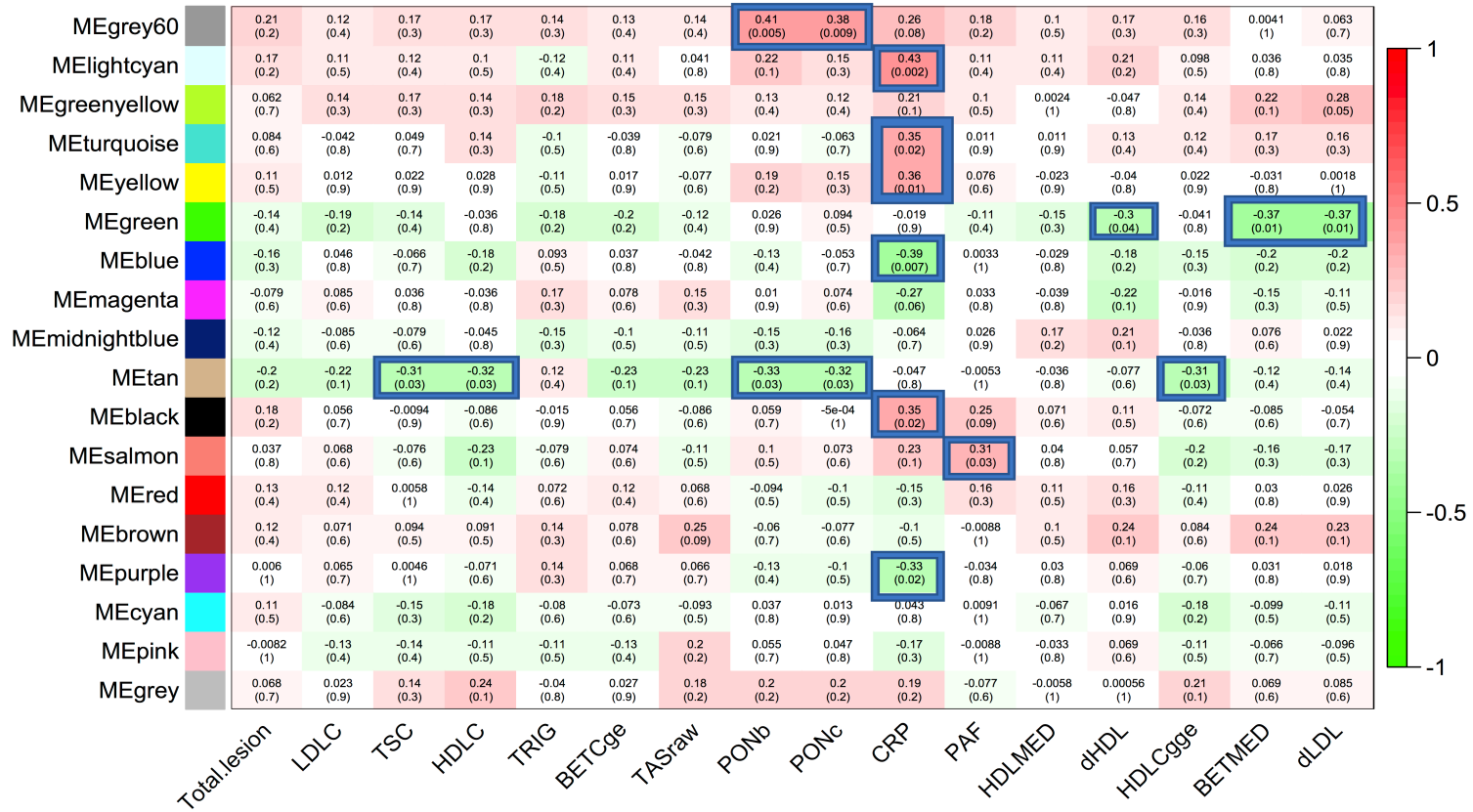

Supplement: S3 Fig — (PDF) [file pone.0271514.s003.pdf]

S4 Fig. Male baseline lesion module trait relationships

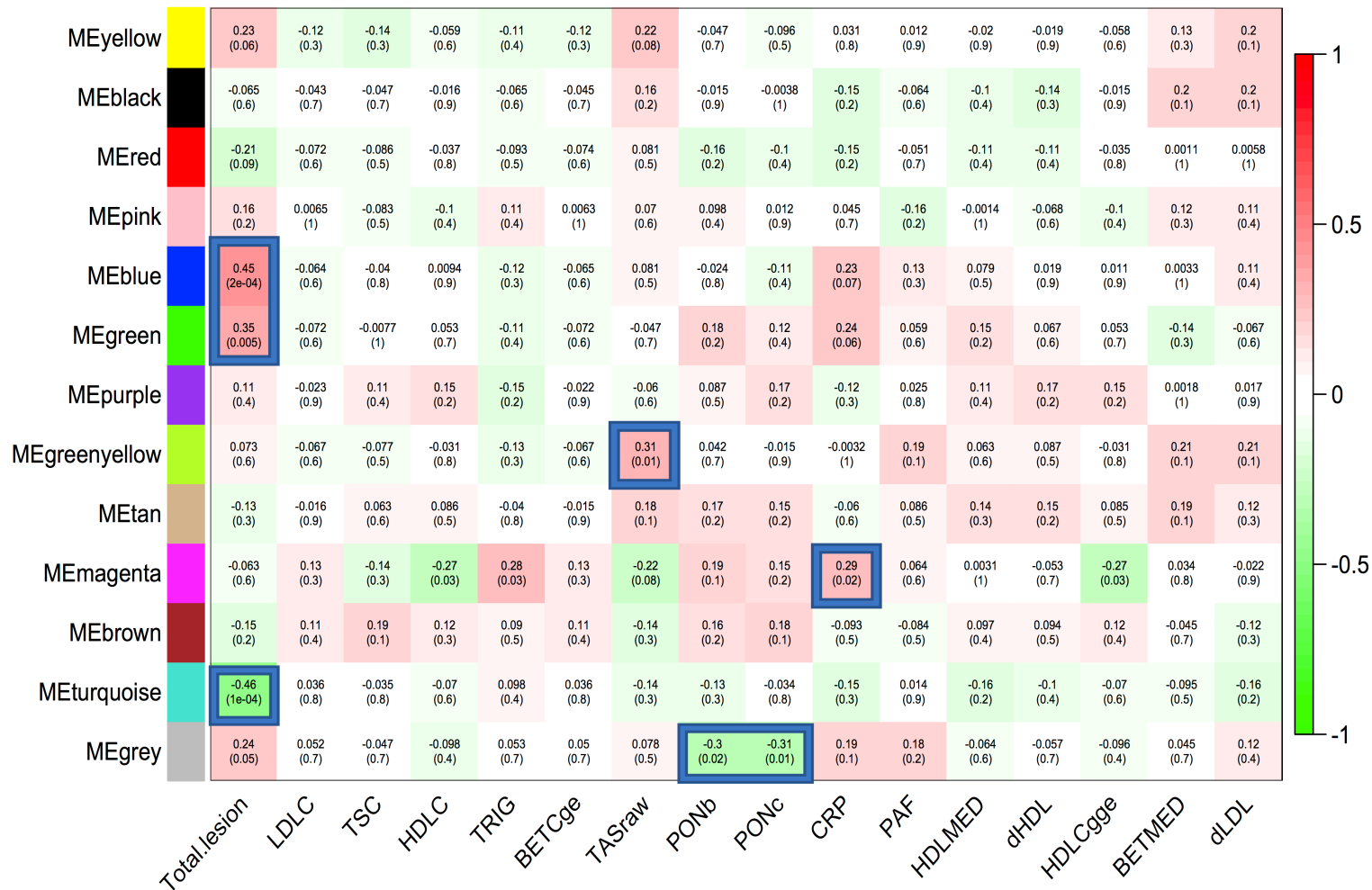

Supplement: S4 Fig — (PDF) [file pone.0271514.s004.pdf]

S5 Fig. Male 7-week lesion module trait relationships

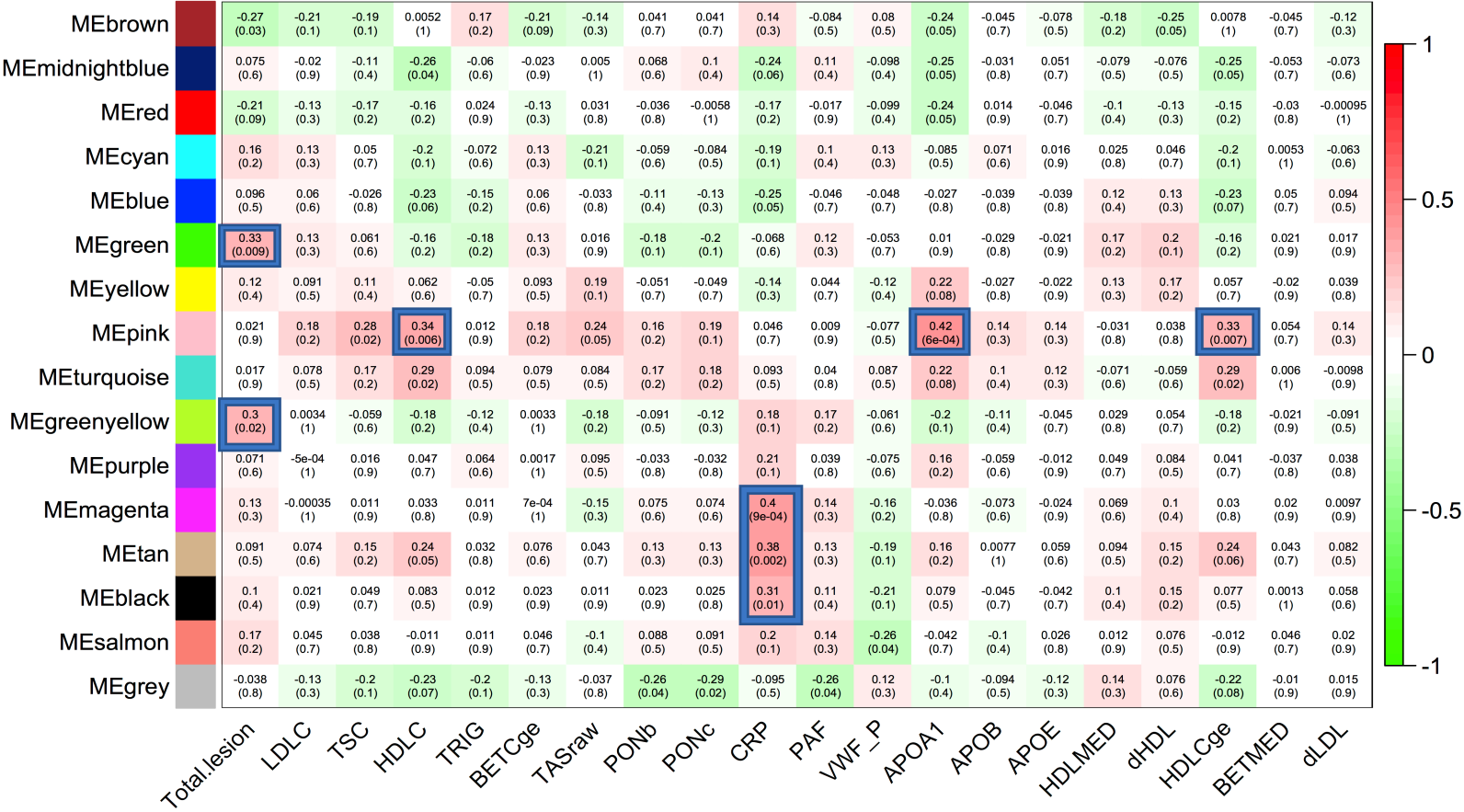

Supplement: S5 Fig — (PDF) [file pone.0271514.s005.pdf]

S6 Fig. Male 2-year lesion module trait relationships

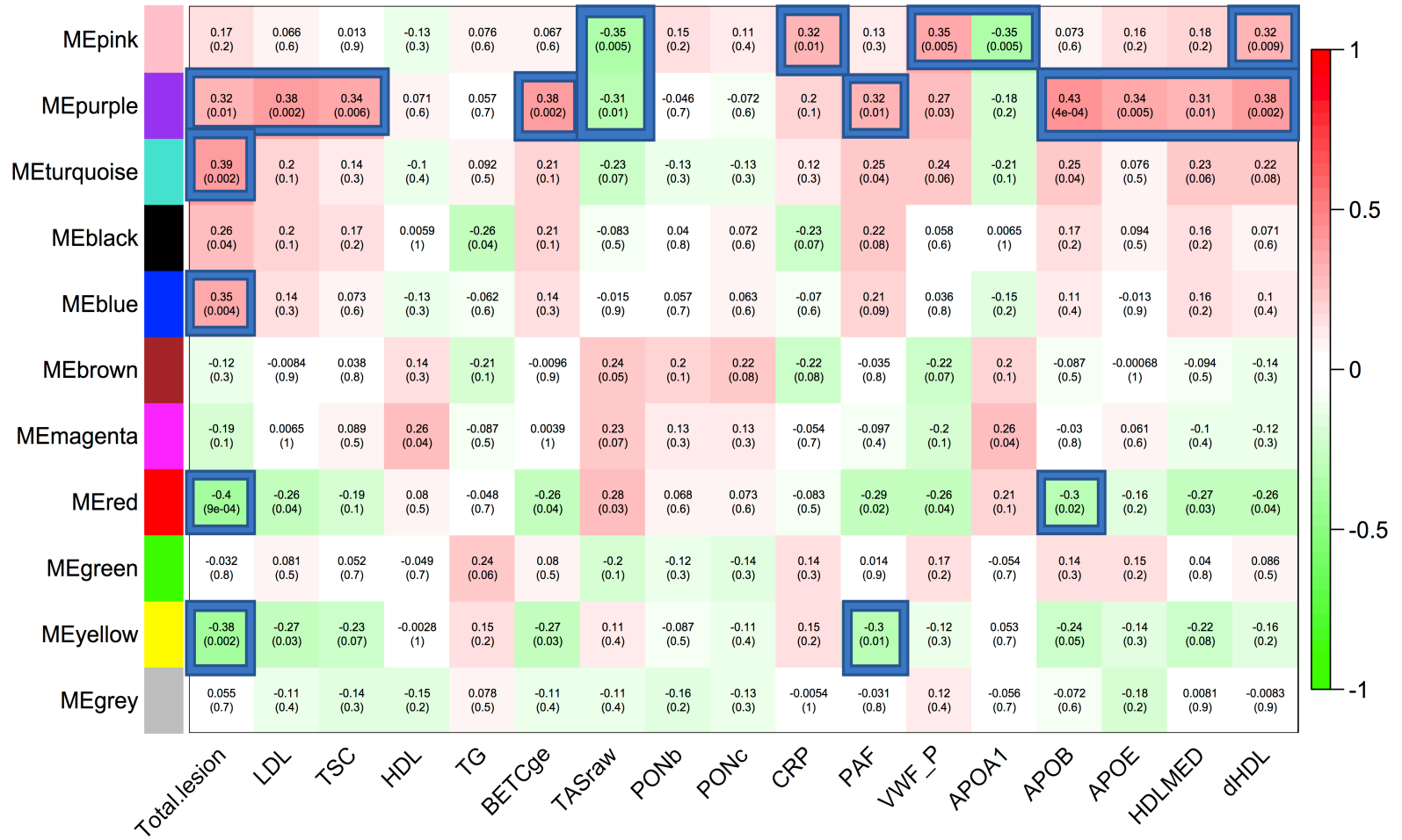

Supplement: S6 Fig — (PDF) [file pone.0271514.s006.pdf]
